# Effects of *Serenoa repens* extract on sexual function in patients with benign prostatic hyperplasia: a systematic review and meta-analysis

**DOI:** 10.1093/sexmed/qfag088

**Published:** 2026-09-16

**Authors:** Ce Dong, Junbin Zheng, Yucheng Jiang

**Affiliations:** Urology, The People’s Hospital of Yuhuan, Taizhou, Zhejiang, 317600, China; Urology, The People’s Hospital of Yuhuan, Taizhou, Zhejiang, 317600, China; Urology, The Second People’s Hospital of Tongxiang, Tongxiang, Zhejiang, 314500, China

**Keywords:** *Serenoa repens* extract, sexual function, quality of life, benign prostatic hyperplasia, meta-analysis

## Abstract

**Introduction:**

*Serenoa repens* extract has been widely used as a phytotherapeutic option for benign prostatic hyperplasia (BPH), potentially offering a milder alternative to pharmaceutical treatments such as 5-alpha reductase inhibitors or alpha-blockers. However, its effects on sexual function remain inconsistent across randomized trials. This study aimed to evaluate the effects of *S. repens* extract on sexual function and quality of life in men withBPH.

**Methods:**

Five electronic databases were searched from inception to January 21, 2026, for randomized controlled trials comparing *S. repens* extract with placebo in men with BPH. The primary outcome was sexual function, with analyses restricted to studies using the International Index of Erectile Function (IIEF) where possible to improve comparability. Quality of life was evaluated as a secondary outcome. Random-effects meta-analyses were performed using standardized mean differences with Hartung–Knapp–Sidik–Jonkman adjustment and restricted maximum likelihood estimation.

**Results:**

Six randomized controlled trials were included. The pooled analysis showed no statistically significant effect of *S. repens* extract on sexual function compared with placebo (Hedges’ *g* = −0.08, 95% CI −0.93 to 0.77; *I*^2^ = 96.16%). The corresponding 95% prediction interval ranged from −2.448 to 2.286, indicating considerable between-study variability and suggesting that future studies could plausibly demonstrate effects favoring either *S. repens* or placebo. In the subgroup analysis restricted to studies using the IIEF, heterogeneity was substantially reduced (Hedges’ *g* = −0.206, 95% CI −0.540 to 0.128; *I*^2^ = 28.76%), although the treatment effect remained statistically non-significant. Similarly, no significant improvement was observed in quality-of-life outcomes, and substantial heterogeneity persisted across studies.

**Discussion:**

Current evidence does not support a clinically meaningful benefit of *S. repens* extract over placebo for improving sexual function or quality of life in men with BPH. Because the overall pooled estimate combined conceptually different sexual function instruments and was associated with substantial heterogeneity, it should be interpreted cautiously. The IIEF-restricted analysis demonstrated markedly lower heterogeneity and may therefore provide a more clinically interpretable estimate of treatment effect, because it was based on a more homogeneous assessment of erectile and sexual function than the overall analysis combining different outcome instruments. The overall certainty of the evidence was very low, and further adequately powered randomized controlled trials using standardized and conceptually comparable sexual function measures are warranted.

**Trial registration:**

This study was registered in the PROSPERO database (CRD420261293450).

## Introduction

Preliminary investigations have indicated that *Serenoa repens* extract, derived from *S. repens*, might offer some advantages in managing symptoms associated with benign prostatic hyperplasia (BPH), including potential influences on sexual function when compared to standard treatments, though the existing studies are limited in scope and rigor, calling for more robust research before any firm recommendations can be made.^1^ The concept of BPH as a condition involving prostate enlargement and related urinary issues was formalized in medical literature decades ago, affecting a notable portion of older men globally. Estimates suggest that among men over 50, prevalence ranges from around 20% to 80% depending on age groups, and it can contribute to broader health concerns when left unaddressed.^2^

In recent times, herbal supplements like *S. repens* extract have emerged as alternative approaches for men experiencing lower urinary tract symptoms (LUTS) linked to BPH. Saw palmetto has been proposed to exert anti-androgenic and anti-inflammatory effects, although the clinical relevance of these mechanisms remains uncertain. This approach could address patient preferences for less invasive methods, such as avoiding reduced libido or erectile challenges often reported with drugs like finasteride, and it might foster better adherence through a more natural profile.^3^

Observational data and initial randomized trials have pointed toward possible links between saw palmetto use and outcomes in BPH, with some noting subtle shifts in sexual parameters like erectile function or overall satisfaction, alongside urinary improvements, in populations with mild-to-moderate symptoms. For instance, certain placebo-controlled studies have observed trends toward fewer sexual adverse events or maintained function in BPH patients, while others highlight variability in physical and quality-of-life measures.^4^ Yet, BPH frequently overlaps with sexual dysfunction, where urinary symptoms may coincide with issues like erectile dysfunction, and effects seen in general BPH groups might not fully translate to those with prominent sexual concerns.

Amid increasing exploration, prior meta-analyses have largely centered on urinary symptoms or general BPH management without uniform focus on sexual function. Many syntheses address LUTS broadly or physical aspects alone, often without applying consistent criteria for sexual outcomes, such as validated scales like the International Index of Erectile Function (IIEF). More recent overviews, including one in 2024, have similarly emphasized urinary scores like the International Prostate Symptom Score (IPSS) with modest effects noted, but they have not systematically isolated sexual function, resulting in pooled results from varied samples that may not align closely with this specific domain.^5^ Previous systematic reviews have reported inconsistent or null effects of saw palmetto for BPH-related symptoms, particularly when analyses were restricted to well-designed placebo-controlled trials, highlighting ongoing uncertainty regarding its clinical effectiveness.^6^

Thus, this review aimed to evaluate whether *S. repens* extract influences sexual function and related quality-of-life outcomes in men with BPH, with particular attention to heterogeneity arising from outcome measurement and formulation differences. The present review was designed to address the following question: Does *S. repens* monotherapy improve sexual function and quality of life compared with placebo in men with BPH?

## Methods

### Eligibility criteria

This review was conducted in accordance with the Preferred Reporting Items for Systematic Reviews and Meta-Analyses (PRISMA) 2020 statement for systematic reviews and meta-analyses, which served as a reporting checklist, and the study protocol was registered in the PROSPERO database (registration number: CRD420261293450).

Eligibility criteria were defined using the PICOS framework. The target population comprised adults diagnosed withBPH.

Population (P): We included adult men diagnosed with BPH, regardless of symptom severity, provided that the diagnosis was clearly stated by the study authors. Studies enrolling mixed populations were eligible only if data for patients with BPH could be extracted separately. Intervention (I): Eligible studies evaluated *S. repens* extract as the sole intervention. Trials using saw palmetto in combination with other herbal preparations, dietary supplements, or pharmacological agents were excluded to ensure that the observed effects could be attributed specifically to *S. repens* extract. Comparator (C): The comparator was required to be placebo. Studies comparing *S. repens* extract with active pharmacological treatments (eg, α-blockers, 5-α reductase inhibitors, or other medications) were excluded, as were head-to-head comparisons without a placebo control. Outcomes (O): The primary outcome was sexual function, assessed using validated instruments (eg, IIEF or comparable scales). Quality of life related to BPH or sexual health was considered a secondary outcome. Studies that did not report any measure of sexual function were excluded. Study design (S): Only randomized controlled trials (RCTs) were eligible. Non-randomized studies, observational studies, case series, reviews, conference abstracts without full data, and animal or in vitro studies were excluded. No restrictions were imposed on publication year or language.

### Exclusion criteria

Studies were excluded if they met any of the following criteria: (1) Sexual function outcomes were not reported, or data were insufficient to calculate effect sizes. (2) The study population included mixed urological conditions (eg, prostatitis, prostate cancer) and results for patients with BPH could not be extracted separately. (3) Duplicate publications or studies reporting overlapping data; in such cases, only the most complete or most recent report was included. (4) Conference abstracts, protocols, or unpublished studies without full-text data available.

### Information sources and search strategy

A comprehensive literature search was conducted and reported in accordance with the PRISMA-S (Preferred Reporting Items for Systematic Reviews and Meta-Analyses–Search Extension) guidelines. The following databases were searched to January 21, 2026: PubMed, Embase, Web of Science Core Collection (WOS), the Cochrane Central Register of Controlled Trials (CENTRAL), and Scopus.

The search strategy was developed and reported in accordance with the PRISMA-S guidelines.^7^

The search strategy combined controlled vocabulary terms (where applicable) and free-text keywords related to three core concepts^1^: BPH and LUTS (eg, “benign prostatic hyperplasia,” “BPH,” “lower urinary tract symptoms,” “LUTS”)^2^; saw palmetto (eg, “saw palmetto,” *S. repens*); and^3^ RCTs (eg, random^*^, trial).

Boolean operators (AND/OR) were used to combine these concepts. Keywords related specifically to sexual function were not included in the search strategy. This decision was made because many RCTs of saw palmetto in patients with BPH do not mention sexual function outcomes in the title or abstract, despite reporting relevant data in the full text. To avoid missing potentially eligible studies, all retrieved records were screened at the full-text level to identify trials reporting sexual function or related quality-of-life outcomes. No restrictions were applied to publication status or language. Clinical trial registries were not searched, as the review focused on published RCTs with available outcome data. The search strategy was initially developed in PubMed and subsequently adapted to the syntax and indexing systems of the other databases. The full search strategies for all databases are provided in Supplementary Appendix 1.

To ensure completeness, the reference lists of all included studies and relevant reviews were manually screened. Completed PRISMA 2020 is provided as Supplementary Appendix 2, and PRISMA-S checklists is provided as Supplementary Appendix 3.

### Study selection

Titles and abstracts were independently evaluated by two reviewers. Full texts of potentially relevant articles were subsequently reviewed to confirm eligibility. Any disagreements were settled through discussion or adjudicated by a third reviewer. EndNote was used to identify and remove duplicate records before the screening process.

### Data extraction

Relevant study characteristics—including the first author’s name, publication year, country, type of saw palmetto extract (phytosterol-enriched or conventional standardized extract), daily dosage (mg/day), presence of sexual dysfunction assessment, intervention group (saw palmetto vs placebo), sample size (*N*), participants’ mean age, baseline IPSS score, baseline sexual function score (eg, Aging Males’ Symptoms [AMS], IIEF, O’Leary score, or Sexual Function Questionnaire [SFQ]), intervention duration (weeks), quality-of-life outcomes, sexual function outcomes, and randomization method—were systematically extracted from each eligibleRCT.

In addition to outcome data, key intervention and study characteristics were extracted, including extract composition (eg, β-sitosterol content), dosage form, capsule composition, daily dose of saw palmetto, dosing frequency, treatment duration, and characteristics of the control intervention where reported. These variables were summarized in tabular form to facilitate qualitative comparison across studies. Data were independently extracted by two reviewers. Any disagreements were resolved through discussion, with arbitration by a third reviewer when necessary. When key information was incomplete or unclear (eg, missing SDs for effect size calculation or absent baseline data), corresponding authors were contacted to request additional details or clarification.

### Risk of bias assessment

The methodological quality of the included studies was independently assessed by two reviewers using Review Manager (RevMan) version 5.4 and STATA 19. Risk of bias 1.0 was evaluated across predefined domains, including sequence generation, allocation concealment, blinding, incomplete outcome data, selective outcome reporting, and other potential biases. Each domain was judged as low, high, or unclear risk in accordance with Cochrane guidelines.

### Effect measures

For each outcome, effect measures were prespecified according to the type of data and measurement instruments used across studies. Sexual function and quality-of-life outcomes were pooled using standardized mean differences (SMDs) because different validated scales were applied (eg, IIEF, AMS, O’Leary score, SFQ). Hedges’ *g* was used to correct for small-samplebias.

For studies in which change-score standard deviations were missing, they were calculated using established formulas assuming a within-group correlation coefficient of 0.5. Sensitivity analyses were conducted by varying the assumed correlation coefficient (*r* = 0.25, 0.5, and 0.75) to evaluate the robustness of the results. The mean and standard deviation (SD) of the change were computed using the following formulas: ${\boldsymbol{Mean}}_{\boldsymbol{change}}={\boldsymbol{Mean}}_{\boldsymbol{pro}}-{\boldsymbol{Mean}}_{\boldsymbol{pre}},{\boldsymbol{SD}}_{\boldsymbol{change}}=\sqrt{{\boldsymbol{SD}}_{\boldsymbol{Pro}}^{\mathbf{2}}+{\boldsymbol{SD}}_{\boldsymbol{Pre}}^{\mathbf{2}}-\mathbf{2}\boldsymbol{r}\ast {\boldsymbol{SD}}_{\boldsymbol{Pre}}\ast {\boldsymbol{SD}}_{\boldsymbol{Pro}}}$. When numerical data were presented only in graphical form, values were extracted using the WebPlotDigitizertool.

### Data synthesis and statistical analysis

Studies were grouped for quantitative synthesis based on intervention characteristics (*S. repens* extract monotherapy vs placebo) and outcome domains (sexual function and quality of life). Only RCTs reporting extractable data for the predefined outcomes were included in the corresponding meta-analyses. Change scores and associated standard deviations were derived when not directly reported, as described above. All conversions followed established methodological guidance for continuous outcomes. Results of individual studies and pooled estimates were summarized in structured tables and visualized using forest plots. Study-specific effect estimates with 95% confidence intervals were displayed, with marker size proportional to study weight. Meta-analyses were conducted using random-effects models with REML estimation, applying the HKSJ adjustment to account for uncertainty due to the small number of studies. Statistical heterogeneity was assessed using Cochran’s *Q* test and quantified using *τ*^2^ and *I*^2^ statistics. Where feasible, 95% prediction intervals were calculated to illustrate the expected range of effects in future settings. All analyses were performed using STATA 19. Prespecified subgroup analyses were performed to explore potential sources of heterogeneity, including sexual function assessment tool (IIEF vs other instruments), geographic region (Asian vs Western populations), treatment duration, and dosage. Robustness of the pooled estimates was evaluated using leave-one-out sensitivity analyses, in which each study was sequentially removed to assess its influence on the overall effect size and heterogeneity.

### Assessment of heterogeneity

Statistical heterogeneity across studies was examined using Cochran’s *Q* statistic and summarized using *τ*^2^ and *τ*, while *I*^2^ was reported as a descriptive measure. To aid interpretation, 95% prediction intervals were generated when feasible to illustrate the potential range of effects in future clinical contexts.

### Reporting bias assessment

Assessment of publication bias was not conducted due to the inclusion of fewer than ten studies, which substantially reduces the power of funnel plot analyses. However, the limited number of trials warrants caution, as the results may still be influenced by unpublished or selectively reported findings.^8^ In addition, clinical trial registries were not searched, so the possibility of missing unpublished studies cannot be excluded.

### Certainty of evidence assessment

The certainty of evidence for each outcome was assessed using the Grading of Recommendations Assessment, Development and Evaluation (GRADE) approach. Evidence from RCTs was initially rated as high certainty and was downgraded based on risk of bias, inconsistency, indirectness, imprecision, and publication bias. The overall certainty of evidence was classified as high, moderate, low, or very low. Assessments were conducted independently by two reviewers, with disagreements resolved through discussion.

## Results

### Study selection

**Figure 1:**
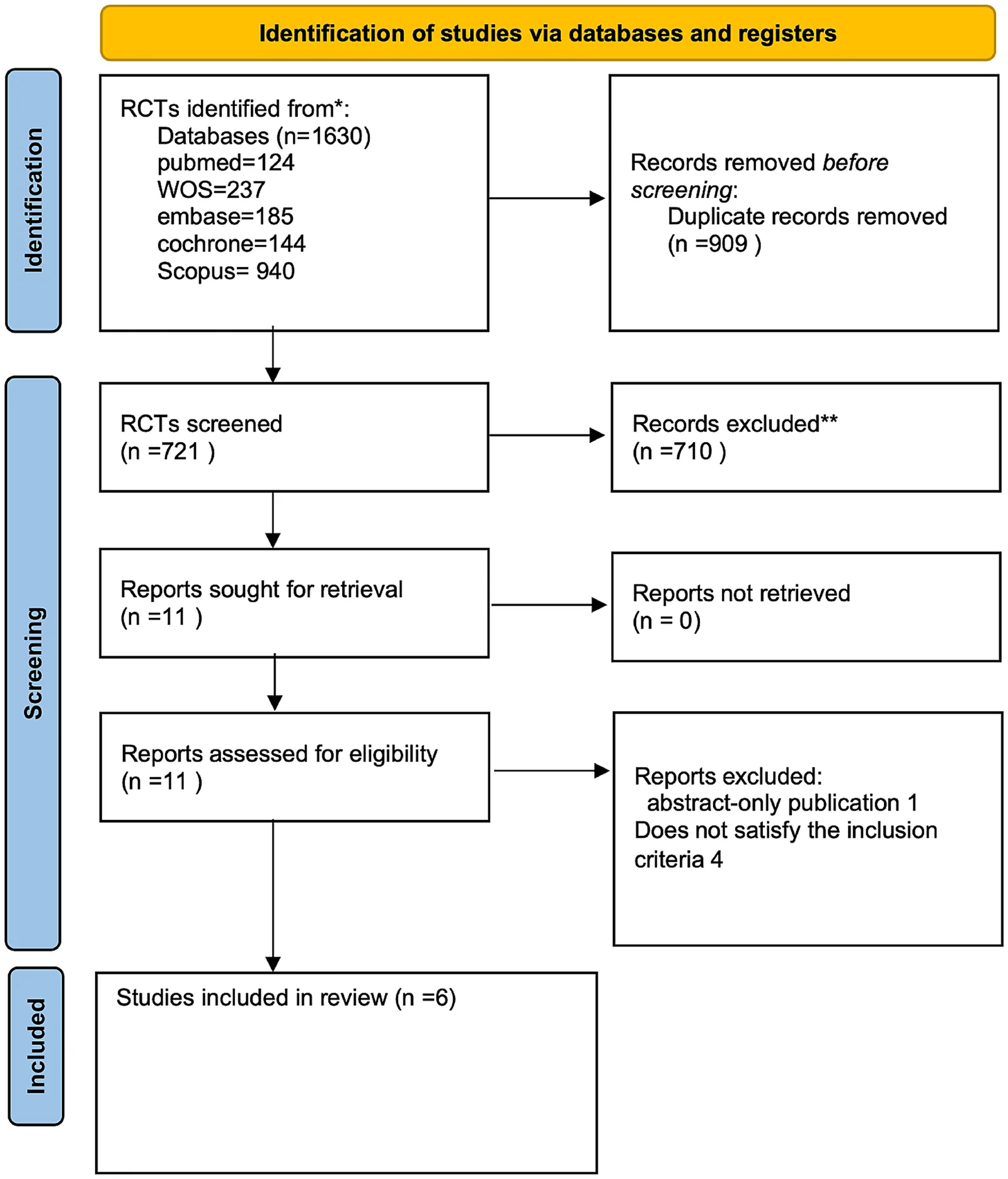
The PRISMA flow diagram. PRISMA, Preferred Reporting Items for Systematic Reviews and Meta-Analyses.

Database searching identified 1630 records from electronic databases, including PubMed (*n* = 124), Web of Science Core Collection (*n* = 237), Embase (*n* = 185), the Cochrane Library (*n* = 144), and Scopus (*n* = 940). After removal of 909 duplicate records, 721 records remained for title and abstract screening, of which 710 were excluded. Eleven reports were sought for retrieval, and all were successfully obtained and assessed for eligibility. Five reports were excluded, including one abstract-only publication and four studies that did not meet the inclusion criteria. Ultimately, six RCTs were included in the systematic review and meta-analysis. The study selection process is illustrated in the PRISMA 2020 flow diagram (Figure 1).

### Characteristics of included studies

A total of six RCTs were included in this meta-analysis. These studies were conducted in multiple countries, including India,^9^ China,^10^ the United States,^1^^,^^4^^,^^11^ and Australia.^3^ The combined sample size across all trials was 1108 participants, with 558 allocated to saw palmetto intervention groups and 550 to placebo control groups. Individual study sample sizes ranged from relatively small single-center trials^3^^,^^4^ to larger multicenter RCTs.^11^

The mean age of participants ranged from the mid-50s to early 60s across studies, with all trials enrolling adult men with LUTS associated with BPH. Baseline symptom severity was generally moderate, with mean IPSS scores ranging from approximately 14 to 21. Sexual function at baseline was assessed using different instruments, including the AMS scale,^9^ the IIEF,^3^^,^^4^^,^^10^ the O’Leary score,^1^ and the SFQ.^4^ All other studies reported change scores directly; for one study in which change scores were not provided, they were calculated assuming a correlation coefficient of 0.5.^3^

Regarding intervention characteristics, five trials evaluated conventional standardized extracts of *S. repens*, while one study investigated a phytosterol-enriched formulation.^9^ Daily dosages of saw palmetto ranged from 200 mg^9^ to 960 mg with dose escalation.^11^ Intervention duration varied from 12 weeks^3^^,^^9^ to 72 weeks,^11^ with intermediate durations of 24-48 weeks reported in other trials. All studies used capsule formulations, and placebo controls consisted of matched inert capsules, most commonly containing maltodextrin.^9^ Detailed study characteristics and intervention protocols are summarized in Tables 1 and 2.

**Table 1:** Main characteristics of the included randomized controlled trials.

Study ID	Year	Country	Focus on phytosterols	Dosage (mg/day)	Sexual dysfunction	Group	*N*	Age (year)	IPSS baseline	Sexual function baseline	Duration time (week)	QoL	Sex function	Randomization method
Sudeep	2020	India	PE	200	Yes	Saw palmetto oil	33	57.76 ± 7.25	21.03 ± 3.86	AMS: 51.52 ± 8.78	12	SF-12: 3.0 ± 1.0	AMS: −1.24 ± 1.84	Randomization number
						Placebo	33	55.18 ± 8.56	21.36 ± 4.54	AMS: 50.85 ± 9.61	12	SF-12: −2.5 ± 1.0	AMS: 0.73 ± 1.26	
Ye	2019	China	CSE	320	Yes	*Serenoa repens*	150	61.47 ± 5.20	18.91 ± 5.48	IIEF: 12.87 ± 6.64	24	IPSS QoL: −1.11 ± 1.27	IIEF: 2.61 ± 11.22	Unclear
						Placebo	154	60.33 ± 6.03	18.27 ± 4.93	IIEF: 12.87 ± 5.79	24	IPSS QoL: 0.64 ± 0.99	IIEF: -0.88 ± 9.72	
Barry	2011	United States	CSE	320 to 960 (escalated)	Yes	Saw palmetto oil	176	61.25 ± 8.72	14.7 ± 5.4	IIEF: 16.5 ± 11.11	72	IPSS QoL: −0.85 ± 0.46	IIEF: −0.52 ± 3.52	Computer-based system
						Placebo	181	60.7 ± 8.08	14.5 ± 5.18	IIEF: 16.0 ± 11.11	72	IPSS QoL: −1.08 ± 0.46	IIEF: −1.06 ± 7.17	
Bent	2006	United States	CSE	320	Yes	Saw palmetto oil	112	62.9 ± 8.0	14.8 ± 4.8	O’Leary: 2.2 ± 1.1	48	SF-36 physical score: 0.10 ± 0.67	O’Leary: −0.06 ± 0.10	Software module
						Placebo	113	63.0 ± 7.4	15.3 ± 5.1	O’Leary: 2.0 ± 1.0	48	SF-36 physical score: −0.51 ± 0.66	O’Leary: 0.07 ± 0.10	
Willetts	2003	Australia	CSE	320	No	*S. repens*	46	62.1 ± 1.2	14.2 ± 0.9	IIEF: 35.8 ± 3.1	12	IPSS QoL: -0.49 ± 2.91^a^	IIEF: 3.61 ± 22.3	Balanced-blocks randomization
						Placebo	47	63.9 ± 1.3	15.1 ± 1.0	IIEF: 31.7 ± 2.9	12	IPSS QoL: -0.69 ± 3.02^a^	IIEF: −0.7 ± 20	
Gerber	2001	United States	CSE	320	Yes	Saw palmetto oil	41	>45	16.7 ± 4.9	SFQ: 10.1 ± 4.7	24	IPSS QoL: −0.7 ± 1.5	SFQ: −0.1 ± 8	Computer number table
						Placebo	44	>45	15.8 ± 4.8	SFQ: 10.5 ± 5.3	24	IPSS QoL: −0.3 ± 1.1	SFQ: −0.1 ± 6.8	

Abbreviations: IPSS, International Prostate Symptom Score; IPSS QoL, IPSS Quality of Life question; QoL, Quality of Life; AMS, Aging Males’ Symptoms scale; IIEF, International Index of Erectile Function; O’Leary score, O’Leary–Sant Interstitial Cystitis Symptom Index; SFQ, Sexual Function Questionnaire; SF-12, 12-Item Short Form Health Survey; SF-36, 36-Item Short Form Health Survey.

a Estimated data extracted from the graph.

**Table 2:** Extraction methods, dosage, and formulations of saw palmetto across included studies.

Study ID	Year	Dose for saw palmetto (mg/day)	Extract type	Group	Composition	Treatment duration (weeks)	Dosage form
Sudeep	2020	200	Supercritical fluid extraction	Saw palmetto (3% β-sitosterol)	Saw palmetto + Maltodextrin	12	Capsule
				Maltodextrin	Maltodextrin	12	Capsule
Ye	2019	320	Hexane extraction	*Serenoa repens* extract	*S. repens* extract	24	Capsule
				Placebo	Placebo	24	Capsule
Gerber	2001	320	Supercritical CO2 extraction	Saw palmetto	Saw palmetto	24	Capsule
				Placebo	Placebo	24	Capsule
Willetts	2003	320	Hexane extract	*S. repens*	*S. repens* extract	12	Capsule
				Placebo	Paraffin oil	12	Capsule
Barry	2011	320 → 960 (escalated)	Proprietary lipidic ethanolic extract	Saw palmetto	Saw palmetto	72	Gelatin
				Placebo	Polyethylene glycol + glycerol	72	Gelatin
Bent	2006	320	Carbon dioxide extract	Saw palmetto extract	Saw palmetto extract	48	Capsules
				Placebo	Polyethylene glycol	48	Capsules

**Table 3:** Sensitivity analysis and subgroup analysis for sexual function.

Leave-one-out sensitivity					
Study omitted	Estimate	[95% Conf.	Interval]	*I* ^2^	Tau^2^
Sudeep 2020	0.13	−0.45	0.72	95.4%	0.4215
Ye 2019	−0.032	−0.82	0.75	95.9%	0.7656
Gerber 2001	−1.00	−0.89	0.69	96.4%	0.7791
Willetts 2003	−0.06	−0.85	0.73	96.3%	0.7745
Barry 2011	−0.08	−0.88	0.72	96.3%	0.7876
Ben 2006	−0.34	−0.73	0.04	95.4%	0.6175
Combined	−0.08	−0.93	0.7 7	96.16%	0.62
Subgroup analysis by tool (IIEF vs others)					
Subgroup (study numbers)	Estimate	[95% Conf.	Interval]	*I* ^2^	Tau^2^
IIEF^3^	−0.206	−0.540	0.128	**28.76**	**0.008**
Other^3^	-	-	-	-	-
Subgroup analysis by race (Asian vs Western)					
Asian^2^	−0.751	−6.472	4.969	89.69	0.365
Western^4^	0.258	−0.867	1.382	94.75	0.474
Subgroup analysis by duration (12 weeks vs >12 weeks)					
Short term^2^	−0.709	−7.273	5.854	88.39	0.472
Long term^4^	0.216	−0.952	1.384	96.58	0.519
Subgroup analysis by dose (300 mg vs other dose)					
300 mg^4^	0.196	−1.006	1.398	95.22	0.548
Other dose^2^	-	-	-	-	-

Bold values indicate low heterogeneity.

### Risk of bias assessment

**Figure 2:**
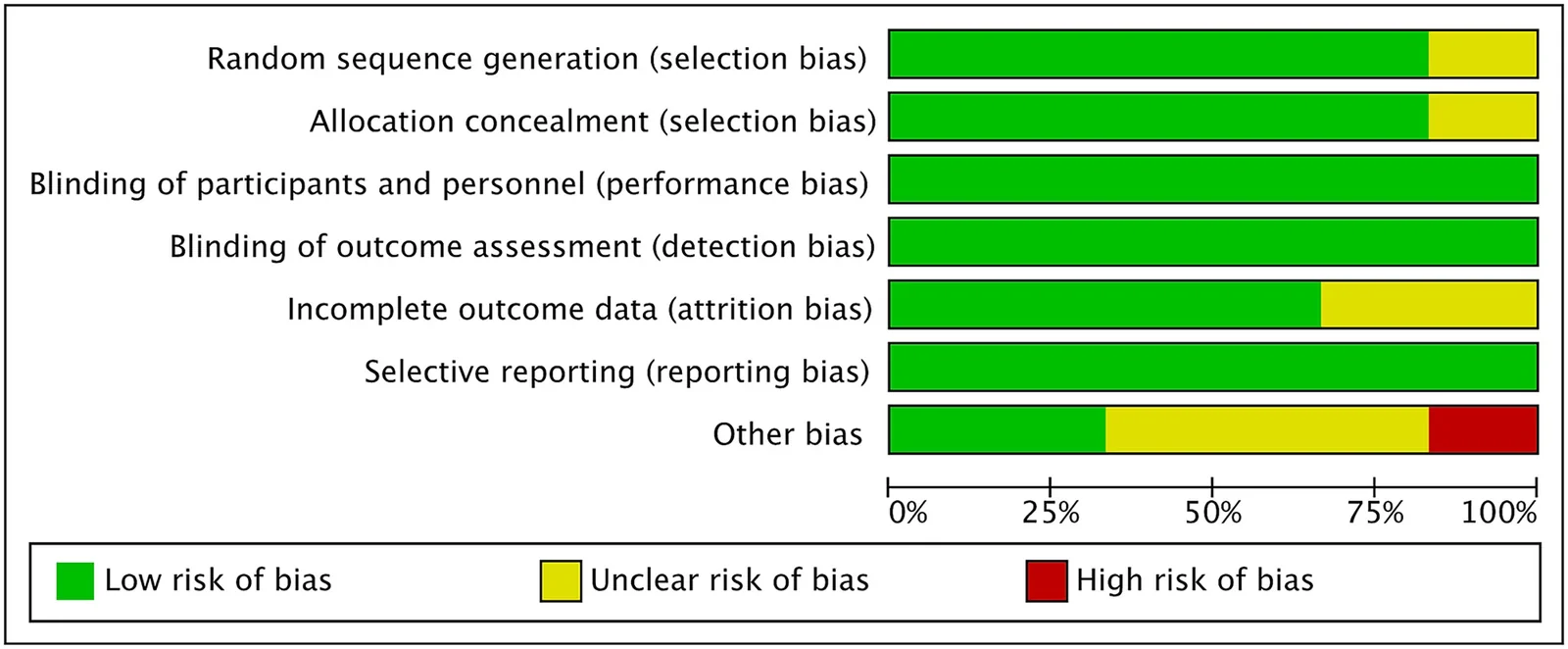
Graph for the risk of bias.

**Figure 3:**
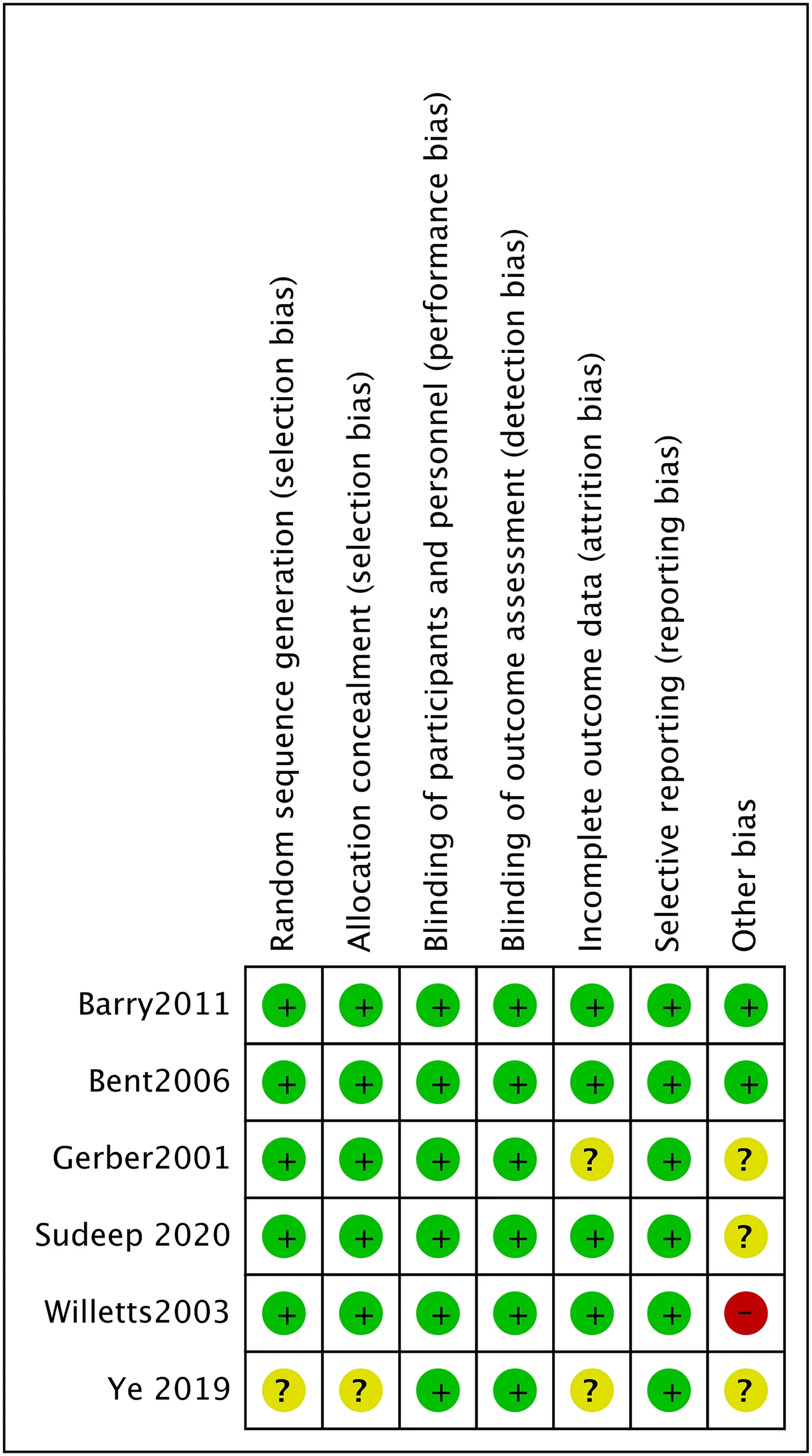
Summary of the risk of bias.

The risk of bias of the included RCTs was assessed using the Cochrane Risk of Bias Tool. Overall, the methodological quality of the included studies was judged to be acceptable. Two large RCTs^1^^,^^11^ demonstrated consistently low risks of bias across all assessed domains, including random sequence generation, allocation concealment, blinding of participants, personnel, and outcome assessors, incomplete outcome data, selective reporting, and other sources of bias. Both studies employed robust randomization procedures and maintained adequate blinding throughout the trial process, resulting in a low overall risk of bias. The results of the risk of bias evaluation are presented as a summary graph (Figure 2) and a detailed table (Figure 3).

Several smaller trials showed greater uncertainty in specific domains due to limited reporting. Ye^10^ did not provide sufficient details regarding random sequence generation or allocation concealment, leading to unclear risk judgments for selection bias–related domains. Similarly, Gerber^4^ demonstrated unclear risk in the domains of incomplete outcome data and other bias, despite otherwise acceptable methodological reporting. The trial by Sudeep et al.^9^ was rated as having an unclear risk of other bias, while remaining domains were assessed as low risk. One study^3^ was judged to have a high risk of other bias because the trial was funded by the product manufacturer.

Across all included studies, blinding of participants and personnel was adequately addressed through double-blind, placebo-controlled designs, resulting in low risk of performance bias. Outcome assessors were blinded in all trials, indicating a low risk of detection bias. Attrition was generally minimal and did not differ substantially between intervention and control groups, and selective outcome reporting was not identified in any of the studies. In summary, the two larger trials^1^^,^^11^ were assessed as having overall low risks of bias, whereas the remaining trials^3^^,^^4^^,^^9^^,^^10^ showed greater uncertainty in specific domains, primarily due to incomplete methodological reporting rather than clear methodological flaws. Such reporting limitations are common in older randomized trials and may reduce confidence in the certainty of evidence, highlighting the importance of transparent reporting and rigorous methodological conduct in future studies.

### Primary outcomes

**Figure 4:**
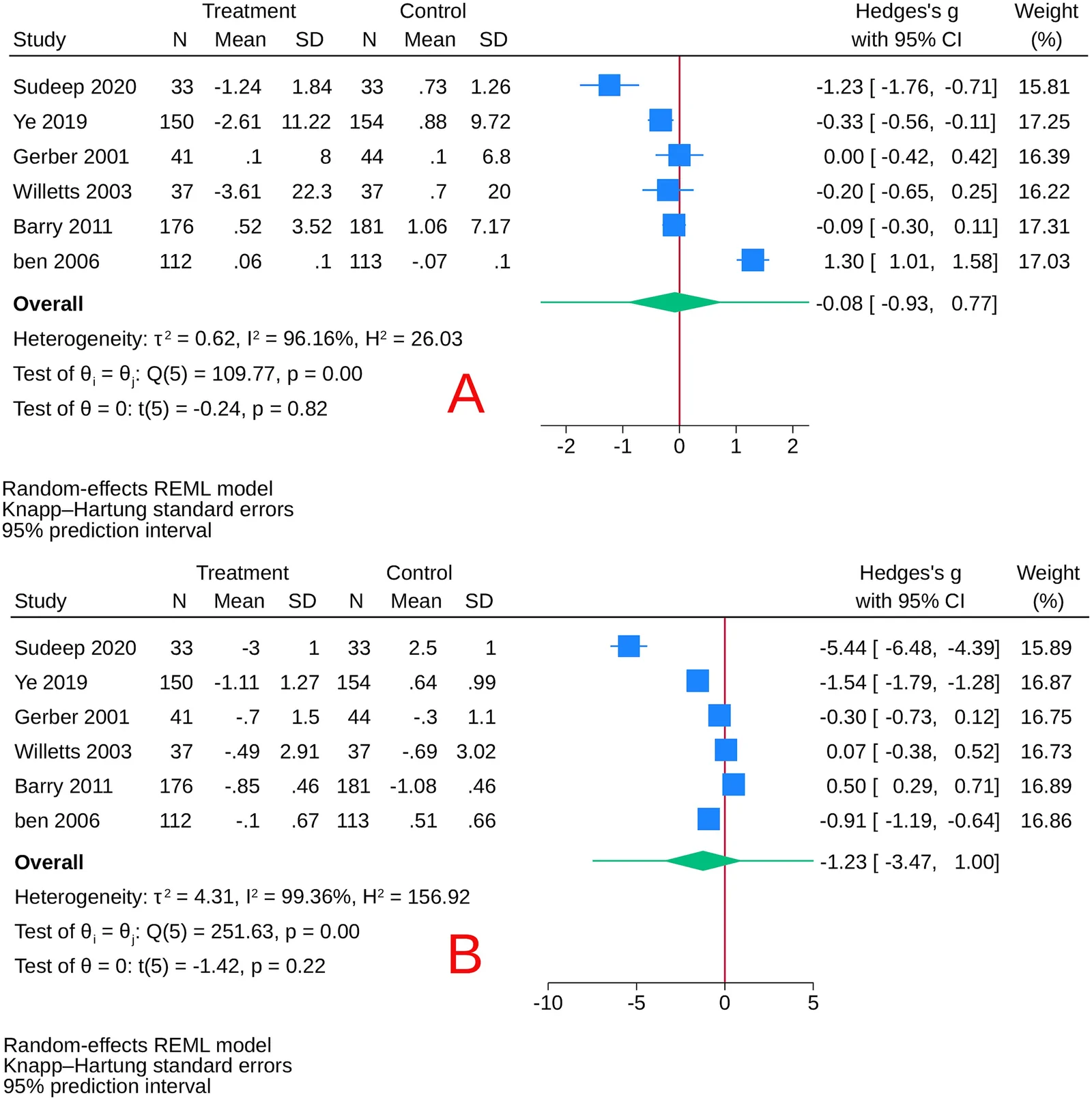
Forest plot of saw palmetto versus placebo (A) for sexual function outcome using a random-effects REML model with Hartung–Knapp adjustment and (B) for quality-of-life outcome using a random-effects REML model with Hartung–Knapp adjustment.

Six RCTs (total *n* = 1108) with generally acceptable methodological quality contributed to the sexual function synthesis, although several studies had unclear risk in specific bias domains. A random-effects meta-analysis was performed using restricted maximum likelihood (REML) estimation with Hartung–Knapp adjustment. The pooled effect estimate did not demonstrate a statistically significant difference between saw palmetto and placebo (Hedges’ *g* = −0.08; 95% CI −0.93 to 0.77; Figure 4A), with substantial between-study heterogeneity observed (*τ*^2^ = 0.62; *I*^2^ = 96.16%; *Q*^5^ = 109.77, *P* < .001). The corresponding 95% prediction interval ranged from −2.448 to 2.286.

Leave-one-out sensitivity analyses showed that the overall effect estimate remained stable and nonsignificant after exclusion of individual studies, indicating no single trial unduly influenced the results, with heterogeneity remaining high throughout (Table 3). Subgroup analyses suggested that outcome measurement tool was a potential source of heterogeneity, as stratification by sexual function instrument markedly reduced heterogeneity in studies using the IIEF (*I*^2^ = 28.76%; *τ*^2^ = 0.008). Among included studies, the IIEF instrument included both IIEF-5 and IIEF-15 versions, which may contribute to residual heterogeneity. In contrast, subgroup analyses by geographic region, treatment duration, and dosage did not meaningfully reduce heterogeneity or change the overall conclusions (Table 3).

### Secondary outcomes

The quality-of-life synthesis included six trials with similar intervention characteristics and overall acceptable risk of bias profiles. Pooled analysis using a random-effects model with REML estimation and Hartung–Knapp adjustment showed no statistically significant improvement in quality of life with saw palmetto compared with placebo (Hedges’ *g* = −1.23; 95% CI −3.47 to 1.00) (Figure 4B).

Substantial between-study heterogeneity was observed (*τ*^2^ = 4.31; *I*^2^ = 99.36%; *Q*^5^ = 251.63, *P* < .001), indicating marked variability in effect estimates across trials. Leave-one-out sensitivity analyses did not identify any influential study, with pooled estimates remaining stable after sequential exclusion of individual trials. Subgroup analyses by assessment tool, dose, duration, and region did not meaningfully explain the observed heterogeneity (Table 4).

**Table 4:** Sensitivity analysis and subgroup analysis for quality oflife.

Leave-one-out sensitivity					
Study omitted	Estimate	[95% Conf.	Interval]	*I* ^2^	Tau^2^
Sudeep 2020	−0.44	−1.15	0.27	98.0%	4.31
Ye 2019	−1.18	−3.24	0.88	97.6%	5.46
Gerber 2001	−1.42	−3.44	0.59	98.4%	5.22
Willetts 2003	−1.50	−3.47	0.47	98.4%	4.96
Barry 2011	−1.58	−3.47	0.30	98.0%	4.31
Ben 2006	−1.30	−3.37	0.76	98.3%	5.46
Combined	−1.23	−3.47	1.00	99.36%	4.31
Subgroup analysis as tool (IPSS vs others)					
Subgroup (study number)	Estimate	[95% Conf.	Interval]	*I* ^2^	Tau^2^
IPSS^4^	−0.32	−1.72	1.08	96.89%	0.76
Other^2^	-	-	-	-	-
Subgroup analysis by dose (300 mg vs other dose)					
300 mg^4^	−0.69	−1.81	0.43	94.17	0.46
Other dose^2^	-	-	-	-	-
Subgroup analysis by duration (12 weeks vs >12 weeks)					
Short term^2^	−2.66	−37.61	32.29	98.89	14.96
Long term^4^	−0.56	−1.95	0.82	97.46	0.74
Subgroup analysis by race (Asian vs Western)					
Asian^2^	−3.45	−28.22	21.32	98.03	7.45
Western^4^	−0.162	−1.131	0.808	93.23	0.35

Sensitivity analyses using assumed within-group correlation coefficients of 0.25, 0.50, and 0.75 yielded nearly identical pooled effect estimates and heterogeneity statistics for both sexual function and quality-of-life outcomes, indicating that the findings were robust to the assumptions used for change-score SD imputation (Table S1).

### GRADE level

According to the GRADE assessment, the certainty of evidence was rated as very low for both sexual function and quality-of-life outcomes. For sexual function, the evidence was downgraded due to considerable between-study heterogeneity, substantial imprecision reflected by wide confidence intervals and the limited number of contributing trials, and some concerns regarding risk of bias arising primarily from incomplete reporting of methodological details in a minority of studies rather than confirmed methodological flaws. For quality of life, similar concerns were identified, with marked heterogeneity and imprecision representing the principal reasons for downgrading. No serious concerns were identified regarding indirectness or publication bias. Overall, the current evidence remains highly uncertain and should be interpreted with caution (Table 5).

**Table 5:** GRADE level.

	Study limitation	Imprecision	Inconsistency	Indirectness	Publication bias	GRADE
Sexual function	Downgraded due to unclear randomization methods in some studies.	Downgraded due to wide confidence intervals and small number of contributing studies.	Downgrade due to high heterogeneity	No downgrade	No downgrade	Very low
QoL	Downgraded due to unclear randomization methods in some studies.	Downgraded due to wide confidence intervals and small number of contributing studies.	Downgrade due to high heterogeneity	No downgrade	No downgrade	Very low

## Discussion

Saw palmetto (*S. repens*) has been widely studied and used as a phytotherapeutic option for BPH–related LUTS.^5^ While early studies suggested potential symptomatic benefits,^12^ subsequent large, well-designed RCTs and systematic reviews have yielded inconsistent or null results.^1^^,^^11^ Consequently, the clinical effectiveness of saw palmetto for BPH remains controversial, warranting further synthesis of evidence across urinary symptoms, sexual function, and quality-of-life outcomes.

In this systematic review and meta-analysis, saw palmetto did not demonstrate a statistically significant benefit over placebo for sexual function, or quality-of-life outcomes. Across all three outcome domains, pooled effect estimates were small, and confidence intervals crossed the null, indicating no clear treatment effect.^1^^,^^3^^,^^11^ These findings are consistent with previous large, well-conducted RCTs that failed to show superiority of saw palmetto over placebo for LUTS-related outcomes.^1^^,^^11^ The absence of statistically significant differences between *S. repens* extract and placebo may partly reflect a substantial placebo effect, particularly for subjective outcomes such as sexual function and quality of life. Patient expectations and the perception of “natural” therapies may attenuate observable differences between intervention and control groups, thereby reducing the apparent treatment effect.

Several factors may account for the lack of statistically significant findings. First, substantial between-study heterogeneity was observed, particularly for sexual function and quality-of-life outcomes. This heterogeneity likely reflects differences in outcome measurement instruments, formulations, dosages, treatment duration, and study populations. One notable finding was the divergent result reported by Bent et al.,^1^ which demonstrated a positive treatment effect despite being one of the largest and methodologically strongest trials. This trial assessed sexual outcomes using the O’Leary questionnaire, whereas the remaining studies primarily used the IIEF, AMS, or SFQ. Although SMDs permit pooling across different validated instruments, these measures may capture related but non-equivalent constructs of sexual health. The O’Leary questionnaire assesses broader symptom- and health-related quality-of-life constructs, whereas the IIEF primarily evaluates erectile and sexual function. Consequently, the overall pooled analysis should be interpreted with caution and may be viewed as an exploratory synthesis, whereas the IIEF-restricted analysis may provide a more clinically interpretable estimate of treatment effect. Therefore, the directionally divergent result reported by Bent et al. is more likely to reflect differences in outcome measurement than differences in methodological quality. Another important source of heterogeneity is the variability in *S. repens* formulations across studies. Included trials used different extraction methods (eg, hexane, CO₂, ethanolic, or phytosterol-enriched extracts), which are not pharmacologically equivalent. This clinical heterogeneity may contribute to inconsistent findings and limit the interpretability of pooled estimates. Future studies should standardize extract formulations to improve comparability. Notably, heterogeneity in sexual function outcomes was markedly reduced when analyses were restricted to studies using the IIEF, suggesting that variability in assessment tools contributed to inconsistent results. However, even within this more homogeneous subgroup, the pooled effect remained nonsignificant, indicating that improved measurement consistency alone does not reveal a clinically meaningful benefit.

Second, imprecision represented a major limitation across outcomes. Several trials were underpowered to detect small-to-moderate effects, and wide confidence intervals indicate considerable uncertainty regarding the true effect size. This limitation was particularly evident for quality-of-life outcomes, which are inherently susceptible to placebo effects and patient expectations, potentially obscuring modest treatment-specific effects.

Third, although most included trials were described as randomized and double-blind, incomplete reporting of methodological details, including random sequence generation and allocation concealment in a minority of studies, limits confidence in the internal validity. These methodological shortcomings contributed to downgrading the certainty of evidence and further weaken the interpretability of pooled estimates.

Recent studies have also explored *S. repens* in combination with conventional pharmacological therapies for BPH. For example, a multicenter randomized study reported potential benefits of *S. repens* combined with alfuzosin compared with alfuzosin alone.^13^ However, such studies were not eligible for the present review because our analysis was restricted to placebo-controlled trials of *S. repens* monotherapy. Therefore, the findings of combination therapy studies should be interpreted separately and cannot be directly compared with the results of the current meta-analysis.

From a clinical perspective, although no significant improvement in sexual function was observed, the absence of deterioration may itself be clinically meaningful. Although no significant deterioration in sexual function was observed in placebo-controlled trials, the current evidence is insufficient to conclude that *S. repens* preserves sexual function. Further comparative studies against pharmacological therapies associated with sexual adverse effects are needed. This characteristic may be valued by patients seeking symptom relief while minimizing treatment-related sexual side effects.

Importantly, these findings do not exclude the possibility that specific formulations, doses, or patient subgroups may derive benefit from saw palmetto. However, such effects remain unproven and cannot be reliably inferred from the current evidence base. Future studies should prioritize rigorous randomization procedures, standardized outcome measures—particularly for sexual function—and sufficient sample sizes to clarify whether any clinically relevant benefit exists.

Overall, the current evidence provides very low-certainty support for the effectiveness of saw palmetto in improving urinary symptoms, sexual function, or quality of life. Similar conclusions have been reported in other meta-analyses in which substantial heterogeneity and imprecision limited confidence in the pooled estimates.^14^ Given the very low certainty of evidence and substantial heterogeneity, no definitive conclusions can be drawn regarding the clinical effectiveness of *S. repens* extract. These findings should therefore be interpreted with caution. In addition, variability in statistical assumptions (eg, correlation coefficients used for change-score estimation) may introduce further uncertainty, although sensitivity analyses suggested that these factors did not materially alter the overall conclusions.

### Limitations

Several limitations of the present review should be acknowledged. First, despite inclusion of several large trials, substantial heterogeneity across studies limited confidence in the pooled estimates. Second, variability in formulations, dosages, treatment duration, and outcome measures may have contributed to inconsistency across outcomes. Third, incomplete reporting of randomization and allocation concealment in some trials introduces potential selection bias and contributed to downgrading the certainty of evidence. Fourth, imprecision remained a key limitation, with wide confidence intervals reflecting limited power to detect small-to-moderate effects. Finally, although saw palmetto appeared to be well tolerated, the absence of consistent efficacy limits conclusions regarding its clinical utility.

This review has additional process-level limitations. We did not search clinical trial registries and could not formally assess publication bias because fewer than 10 studies were available, so missing or selectively reported results cannot be excluded.

In addition, some outcomes required data conversion (eg, deriving change-score SDs with an assumed correlation and digitizing values from figures), which may introduce additional uncertainty. Additional uncertainty arises from the assumptions used in deriving change-score standard deviations, although sensitivity analyses suggested that these assumptions did not materially alter the overall conclusions.

## Conclusion

This systematic review and meta-analysis did not identify a statistically significant benefit of *S. repens* extract over placebo for sexual function or quality of life in men with BPH. However, given substantial heterogeneity, imprecision, and very low certainty of evidence, these findings should be interpreted with caution. Further high-quality trials using standardized outcome measures and well-defined formulations are needed.

## Supplementary Material

Supplementary_material_qfag088

## Data Availability

The data extracted from the included studies and used in all analyses are provided within the article and its supplementary materials. The PRISMA 2020 and PRISMA-S checklists, along with the full search strategies, are available as supplementary files. Analytic code was not publicly shared but is available from the corresponding author upon reasonable request.
