# Supplementary material for: Effects of Serenoa repens extract on sexual function in patients with benign prostatic hyperplasia: a systematic review and meta-analysis
Source: Sex Med. 2026 Sep 16;14(6):qfag088. doi: 10.1093/sexmed/qfag088 (PMC13580080; doi:10.1093/sexmed/qfag088)
Supplement: Supplementary file 1 [file supplementary_material_qfag088.zip › appendix 3.pdf]

Section/topic	#	Checklist item	Location(s) Reported
**INFORMATION SOURCES AND METHODS**			
Database name	1	Name each individual database searched, stating the platform for each.	PubMed, Embase, Web of Science Core Collection, cochrane, Scopus
Multi-database searching	2	If databases were searched simultaneously on a single platform, state the name of the platform, listing all of the databases searched.	Not applicable
Study registries	3	List any study registries searched.	Not searched
Online resources and browsing	4	Describe any online or print source purposefully searched or browsed (e.g., tables of contents, print conference proceedings, web sites), and how this was done.	Methods – Information sources and search strategy
Citation searching	5	Indicate whether cited references or citing references were examined, and describe any methods used for locating cited/citing references (e.g., browsing reference lists, using a citation index, setting up email alerts for references citing included studies).	Methods – Information sources and search strategy
Contacts	6	Indicate whether additional studies or data were sought by contacting authors, experts, manufacturers, or oth	Not conducted
Other methods	7	Describe any additional information sources or search methods used.	Not applicable
SEARCH STRATEGIES			
Full search strategies	8	Include the search strategies for each database and information source, copied and pasted exactly as run.	Supplementary Appendix 1
Limits and restrictions	9	Specify that no limits were used, or describe any limits or restrictions applied to a search (e.g., date or time pe	Methods – Information sources and search strategy
Search filters	10	Indicate whether published search filters were used (as originally designed or modified), and if so, cite the filte	Not used
Prior work	11	Indicate when search strategies from other literature reviews were adapted or reused for a substantive part or	Not applicable
Updates	12	Report the methods used to update the search(es) (e.g., rerunning searches, email alerts).	Methods – Information sources and search strategy
Dates of searches	13	For each search strategy, provide the date when the last search occurred.	Methods – Information sources and search strategy
PEER REVIEW			
Peer review	14	Describe any search peer review process.	Not conducted
MANAGING RECORDS			
Total Records	15	Document the total number of records identified from each database and other information sources.	Results – Study selection (PRISMA flow diagram)
Deduplication	16	Describe the processes and any software used to deduplicate records from multiple database searches and o	Methods – Study selection

PRISMA-S: An Extension to the PRISMA Statement for Reporting Literature Searches in Systematic Reviews
Rethlefsen ML, Kirtley S, Waffenschmidt S, Ayala AP, Moher D, Page MJ, Koffel JB, PRISMA-S Group.
Last updated February 27, 2020.
