# Supplementary material for: Effects of Serenoa repens extract on sexual function in patients with benign prostatic hyperplasia: a systematic review and meta-analysis
Source: Sex Med. 2026 Sep 16;14(6):qfag088. doi: 10.1093/sexmed/qfag088 (PMC13580080; doi:10.1093/sexmed/qfag088)
Supplement: Supplementary file 1 [file supplementary_material_qfag088.zip › supplementary table 1.docx]

Supplementary table 1. Sensitivity analyses using different assumed within-group correlation coefficients for change-score SD imputation

| **Outcomes** | **Assumed correlation** | **Hedges' g** | [95% Conf. | Interval] | **I²** |
| --- | --- | --- | --- | --- | --- |
| sex function | r = 0.25 | -0.062 | -0.910 | 0.787 | 96.14 |
|  | r = 0.50 | -0.081 | -0.931 | 0.770 | 96.16 |
|  | r = 0.75 | -0.064 | -0.912 | 0.785 | 96.14 |
| QoL | r = 0.25 | -1.233 | -3.466 | 1.000 | 99.36 |
|  | r = 0.50 | -1.232 | -3.466 | 1.001 | 99.36 |
|  | r = 0.75 | -1.232 | -3.465 | 1.002 | 99.36 |

QoL: Quality of Life.
