# Supplementary material for: Effects of Serenoa repens extract on sexual function in patients with benign prostatic hyperplasia: a systematic review and meta-analysis
Source: Sex Med. 2026 Sep 16;14(6):qfag088. doi: 10.1093/sexmed/qfag088 (PMC13580080; doi:10.1093/sexmed/qfag088)
Supplement: Supplementary file 1 [file supplementary_material_qfag088.zip › appendix 1.docx]

search from inception to January 21, 2026

Pubmed

Search strategy: (((((benign prostatic hyperplasia[Title/Abstract]) OR (LUTS[Title/Abstract])) OR (BPH[Title/Abstract])) OR (Lower Urinary Tract Symptoms[Title/Abstract])) AND ((saw palmetto[Title/Abstract]) OR (Serenoa repens[Title/Abstract]))) AND ((random*[Title/Abstract]) OR (trial[Title/Abstract]))

Scopus

Search strategy: TITLE-ABS-KEY ( random* OR trial ) AND ( "benign prostatic hyperplasia" OR "LUTS" OR "BPH" OR "Lower Urinary Tract Symptoms" ) AND ( "saw palmetto" OR "Serenoa repens" )

Embase:

Search strategy: ('benign prostatic hyperplasia':ti,ab,kw OR 'Lower Urinary Tract Symptoms':ti,ab,kw OR luts:ti,ab,kw OR bph:ti,ab,kw)

AND

(random*:ti,ab,kw OR trial:ti,ab,kw)

AND

('saw palmetto':ti,ab,kw OR 'Serenoa repens':ti,ab,kw)

Cochrane

Search strategy:

#45 (“benign prostatic hyperplasia”):ti,ab,kw

OR ("Lower Urinary Tract Symptoms"):ti,ab,kw

OR (bph):ti,ab,kw

OR (luts):ti,ab,kw

#46 ("saw palmetto"):ti,ab,kw

OR ("Serenoa repens"):ti,ab,kw

#47 #45 AND #46

Web of Science

Search strategy:

TS=(

"benign prostatic hyperplasia"

OR luts

OR "Lower Urinary Tract Symptoms"

OR bph

)

AND

TS=(

"saw palmetto"

OR “Serenoa repens”

)

AND

TS=(

random*

OR trial

)
